# Sustained posterior contralateral activity indicates re-entrant target processing in visual change detection: an EEG study

**DOI:** 10.3389/fnhum.2014.00247

**Published:** 2014-05-05

**Authors:** Daniel Schneider, Sven Hoffmann, Edmund Wascher

**Affiliations:** Leibniz Research Centre for Working Environment and Human FactorsDortmund, Germany

**Keywords:** attention, perception, short-term memory, N2pc, SPCN, re-entrant processing

## Abstract

The present study investigated the neural mechanisms that contribute to the detection of visual feature changes between stimulus displays by means of event-related lateralizations of the electroencephalogram (EEG). Participants were instructed to respond to a luminance change in either of two lateralized stimuli that could randomly occur alone or together with an irrelevant orientation change of the same or contralateral stimulus. Task performance based on response times and accuracy was decreased compared to the remaining stimulus conditions when relevant and irrelevant feature changes were presented contralateral to each other (contralateral distractor condition). The sensory response to the feature changes was reflected in a posterior contralateral positivity at around 100 ms after change presentation and a posterior contralateral negativity in the N1 time window (N1pc). N2pc reflected a subsequent attentional bias in favor of the relevant luminance change. The continuation of the sustained posterior contralateral negativity (SPCN) following N2pc covaried with response times within feature change conditions and revealed a posterior topography comparable to the earlier components associated with sensory and attentional mechanisms. Therefore, this component might reflect the re-processing of information based on sustained short-term memory representations in the visual system until a stable target percept is created that can serve as the perceptual basis for response selection and the initiation of goal-directed behavior.

## Introduction

According to the biased competition account of visual selective attention, goal-directed behavior in a rich visual environment depends on the representation of relevant visual signals in higher-level executive instances that are capable to process only a strongly limited amount of information. The biased competition account suggests that the strongest represented signals prevail in competitive visual processing and are therefore represented in highler-level areas (Desimone and Duncan, 1995; Chelazzi et al., 2001; Everling et al., 2002). The outcome of competitive processing is determined both by the physical characteristic of presented information and by intentions of the observer that bias information processing in the visual system toward relevant signals.

Furthermore, information processing is not implemented in an exclusively unidirectional way with slow competitive interactions ranging from sensory to higher-level structures. It rather consists of iterative circuits between and within sensory and higher-level areas that can be differentiated into the feed-forward stream and the re-entrant processing of information (Lamme and Roelfsema, 2000). Re-entrant signals from higher-level areas are automatically sent back to lower areas when the feed-forward processing stream reaches a certain stage. This drawing on information in sensory areas might be required for evaluating the active perceptual hypothesis in the context of behavioral goals and might therefore be required for guiding the initiation of goal directed behavior (Di Lollo et al., 2000; Lamme and Roelfsema, 2000; Fahrenfort et al., 2008).

The present study made use of event-related potentials (ERPs) of the EEG that were shown to be indicative of such feedback or re-entrant mechanisms (see Robitaille and Jolicoeur, 2006; Schneider and Wascher, 2013) and studied potential correlations with behavioral performance in a visual change detection task (see Wascher and Beste, 2010; Schneider et al., 2012a,b). The first ERP response to visual feature changes in stimuli presented on the horizontal meridian is a posterior positivity (change-related posterior positivity) that appears with a varying latency of about 100 ms subsequent to change presentation (depending on the changing feature; Kimura et al., 2005, 2006). The component was larger at contralateral compared to ipsilateral posterior electrodes when a lateralized feature change was presented in a stimulus display (Schneider et al., 2012a,b) and might be attributed to the dishabituation of neurons encoding the changing stimulus compared to those encoding contralateral non-changing signals (Luck and Hillyard, 1994a; Verleger et al., 2012).

Subsequent to this posterior positivity, lateralized feature changes trigger multiple posterior asymmetric deflections in the ERP that are not specific to stimulus change and are caused by an increase in negativity contralateral to the most salient or currently relevant stimulus. While the posterior contralateral negativity in the N1 time window (N1 posterior contralateral or N1pc; 150–200 ms after stimulus presentation) is based on the physical characteristics of presented stimuli (Shedden and Nordgaard, 2001; Wascher and Beste, 2010), N2pc appears with a peak latency of about 250 ms subsequent to stimulus presentation and is associated with selective attentional processing of relevant stimuli against lateral distracting information (Luck and Hillyard, 1994a,b; Eimer, 1996; Wascher and Wauschkuhn, 1996). A later posterior contralateral negativity labeled sustained posterior contralateral negativity (SPCN; Robitaille and Jolicoeur, 2006) is associated with the drawing on visual short-term memory (vSTM) or working memory representations required for further cognitive operations with the stimulus material (Prime et al., 2011; Mazza and Caramazza, 2012). SPCN might be based on the same neural mechanisms leading to the contralateral delay activity (CDA) in tasks with long intervals between stimulus displays that directly demand the retention of visuo-spatial information in vSTM or working memory (see Vogel and Machizawa, 2004). Compared to this, vSTM representations in tasks that require an immediate choice reaction to briefly presented stimuli (reflected by SPCN) might serve as a stable template for evaluating if perceptual information corresponds to current response tendencies or behavioral goals. This evaluation should critically depend on re-entrant connections between higher-level executive instances and lower-level sensory areas. Accordingly, SPCN continuation was linked to the time required for responding to target stimuli (Robitaille and Jolicoeur, 2006), while no SPCN was shown when participants failed to report the presence of relevant visual signals (Prime et al., 2011).

In the present study, participants were instructed to locate a relevant luminance change in two lateralized bars that was randomly presented simultaneous with an irrelevant orientation change of the same or contralateral bar (Wascher and Beste, 2010; Schneider et al., 2012b). When an orientation change was presented contralateral to the less salient luminance change (contralateral distractor condition), response times (RTs) and detection accuracy were deteriorated compared to unilateral feature change conditions. N1pc reflected the response to the lateralized feature changes based on stimulus saliency and N2pc was associated with selective attentional focusing on the relevant stimulus. SPCN^1^ was associated with an iterative target analysis required for change localization and was larger in amplitude when target discrimination was complicated by the contralateral distractor compared to unilateral change conditions (Wascher and Beste, 2010; Schneider et al., 2012b; Schneider and Wascher, 2013). We further hypothesize that the continuation of this late posterior contralateral negativity should be modulated by the time required for change localization (see Robitaille and Jolicoeur, 2006). Accordingly, a later return of SPCN to baseline should be observed in the contralateral distractor condition compared to the unilateral change conditions.

However, if SPCN is indeed associated with sustained representations in vSTM required for evaluating if selected visual information corresponds to current response tendencies or behavioral goals, this should also be indicated by a co-variation of its continuation with differing RTs within stimulus conditions. Therefore we defined 10 bins of trials within each change condition that were arranged according to the time required for responding to the location of the relevant luminance change. We hypothesize that a longer lasting pattern of posterior asymmetric activation in favor of the relevant luminance change is shown for slow compared to fast response trials. Additionally, if the mentioned posterior asymmetries associated with sensory and attentional processing (i.e., change-related positivity, N1pc, N2pc) and SPCN reveal comparable posterior topographies (see Robitaille and Jolicoeur, 2006; Brisson and Jolicoeur, 2008), this would support the notion that these mechanisms rely on the same neural structures and thus an iterative analysis of information in posterior visual areas is required to form a stable representation of relevant feature changes in vSTM.

## Materials and methods

### Participants

12 participants (seven female) with an age between 20 and 30 years (*M* = 25.2, *SD* = 2.8) took part in the experiment. All participants were right handed. As reported in a screening questionnaire, none of the participants reported any known neurological or psychiatric diseases and had normal or corrected-to-normal vision. They took part in return for course credit or a payment of 8€ per hour and provided informed written consent prior to beginning the experiment. The local ethics committee approved the study.

### Stimuli and procedure

**Figure 1:**
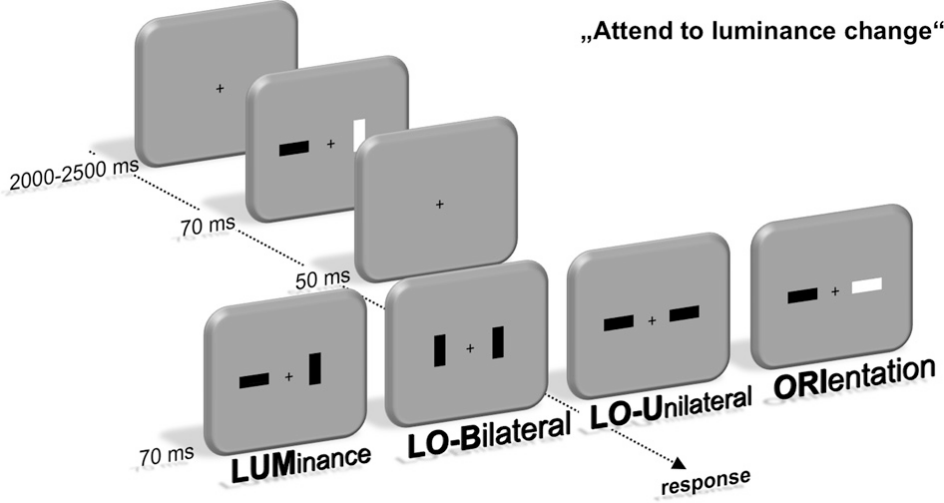
**Experimental setup**. Participants were instructed to localize a change of luminance within a fast sequence of two stimulus displays containing two lateralized bars. These luminance changes either occurred alone (LUM) or simultaneous with an orientation change of the same (LOU) or contralateral bar (LOB). Additionally, only the orientation of one bar changed in one out of four trials (ORI). Localization was accomplished by a button press at the side of the luminance change using the index finger.

Participants were seated in front of a 22-inch CRT monitor (viewing distance 120 cm, 100 Hz) in a dimly lit chamber. Stimulus presentation was controlled by a VSG 2/5 graphic accelerator (Cambridge Research Systems, Rochester, UK). In the first stimulus display, two bars with an area of 0.76 cm^2^ and a 1:2.41 length-to-width ratiowere presented 1.1° lateral to a central fixation cross. Their luminance was either brighter (45 cd/m^2^) or darker (20 cd/m^2^) than the background (30 cd/m^2^), leading to a constant Michelson contrast of 0.2 to keep stimulus saliency comparable between dark and bright stimuli (Michelson, 1927). Furthermore, the bars were presented in a horizontal or vertical orientation. This first stimulus display lasted for 70 ms and was followed by a 50 ms interval with only the fixation cross present. Afterwards, a second display with basically the same layout was presented for 70 ms that contained four different kinds of feature changes between stimulus display 1 and 2 (see Figure 1). The luminance (LUM) or orientation (ORI) of one bar could change. Additionally, luminance and orientation changes were presented simultaneously at the same bar (LOU—luminance and orientation unilateral) or contralateral to each other (LOB—luminance and orientation bilateral).

Participants were instructed to respond by pressing a key with their left or right index finger on the side of the relevant luminance change. The orientation change was declared as irrelevant and was not predictive for the location of the luminance change. Accordingly, all trials only containing an irrelevant orientation change (ORI) were no-go trials. Participants were instructed to respond as quickly and accurately as possible. The characteristics of all bars in the first and second stimulus display were randomly intermixed to assure that the observer could not anticipate a luminance or orientation change on basis of the first stimulus display and thus had to process the feature change for solving the task. The intertrial interval varied between 2000 and 2500 ms (uniform distribution). The experiment was divided into eight blocks with 384 trials each. A 3-min break followed after each block to counteract confounding effects of fatigue during the experiment. This led to an overall experimental time of about 3 h. Compared to prior experiments with the same experimental setup (Wascher and Beste, 2010; Schneider et al., 2012a,b; Schneider and Wascher, 2013), short presentation times (70 ms for both the first and second stimulus display) were used to induce only brief visual input of the feature changes and thus potentially increase the need for prolonged target processing in order to solve the task.

### Data analysis

#### Behavioral data

Responses were recorded from presentation of the first stimulus display up to 1200 ms after onset of the second display. Responses prior to 150 ms after the second display were categorized as “fast guesses.” Error trials involved missed responses (no response in the 1200 ms interval), fast guesses, responses at the wrong stimulus side and misplaced responses in the ORI no-go trials. The experimental effects on error rates were quantified by an analysis of variance (ANOVA) with the within-subject factors *change condition* (LUM, LOU, LOB, ORI) and *stimulus side* (left vs. right). The same ANOVA setup was used for RTs. Yet, only three change conditions (LUM, LOU, LOB) were entered into the analysis, because the ORI condition was a no-go condition. Benjamini-Yekutieli correction was used for pairwise comparisons (Benjamini and Yekutieli, 2001).

#### EEG data

EEG was recorded from 60 Ag/AgCl active electrodes (ActiCap, Brain Products, Gilching, Germany) affixed across the entire scalp according to the extended 10/20 System (Pivik et al., 1993). Eye movements were recorded continuously from two electrode pairs affixed above and below the left eye (vertical EOG) and at the outer canthi of each eye (horizontal EOG). EEG and EOG were sampled on-line with a frequency of 1 kHz by a BrainAmp DC-amplifier with a 250 Hz low-pass filter. For ERP analyses, segments with a length of 1520 ms (320 ms before to 1200 ms after the change-relevant second stimulus display) were defined for further processing. Baseline was set to the 200 ms interval preceding the first stimulus display (i.e., −320 to −120ms referred to the change display). Only correct responses were used for ERP analyses. Segments were checked offline for artifacts (zero-lines, fast shifts, or drifts) and the remaining influence of ocular artifacts upon EEG activity was corrected by the algorithm proposed by Gratton et al. (1983). Subsequently, EEG data were transformed via current source density (CSD) interpolation. The CSDs were calculated according to Perrin et al. (1989) using the CSD-toolbox (Kayser and Tenke, 2006a,b) and EEGLAB (Delorme and Makeig, 2004) for Matlab^©^. The advantage of the CSD transformation compared to standard ERPs is that the CSD transformed data are reference-free spatially enhanced reflections of the location and intensity of ERP sources (Mitzdorf, 1985), in other words: the CSD acts as a spatial filter that removes the effect of remote sources to local surface potentials (compare e.g., Vidal et al., 2003; Roger et al., 2010).

#### Event-related lateralizations (ERLs)

Posterior ERLs (PO7/PO8) were calculated from the ERPs of the CSD transformed EEG by subtracting the activation ipsilateral from the activation contralateral to a laterally presented stimulus. This difference wave served for addressing the visuospatial processing of the presented feature changes and provides valuable information about the contralateral vs. ipsilateral processing preference in the visual system (Wascher and Wauschkuhn, 1996; Wolber and Wascher, 2003; Verleger et al., 2012). In case of the LOB condition, the side of the luminance change served as the spatial reference.

The posterior ERLs for each change condition revealed multiple deflections in the course of visual processing that were classified according to their temporal occurrence and polarity (i.e., change-related positivity, N1pc, N2pc, SPCN). Mean amplitudes in a 20 ms interval oriented at the peak in the grand averaged ERL were used as indicators of these posterior asymmetries (see Figure 3A). Their reliable occurrence was tested by means of *t*-tests against zero. Additionally, separate ANOVAs for each component were run with the within-subject factor change condition to investigate processing differences between stimulus conditions. Uncorrected *F*-values, Greenhouse-Geisser epsilon (ε), corrected *p*-values (if degrees of freedom were >1; see Vasey and Thayer, 1987) and partial eta squared (η^2^_*p*_) are reported for all ANOVAs concerning behavioral and EEG data. Benjamini-Yekutieli corrected paired samples *t*-tests were run when pairwise comparisons were applicable (Benjamini and Yekutieli, 2001). Uncorrected *t*-values, corrected *p*-values and Cohen's d are reported for all *t*-tests.

#### ERL vincentiles

Since ERLs cannot be obtained in single-trial EEG data (multiple trials are required for subtracting ipsilateral from contralateral activity referred to a lateralized visual event), and we were interested in the co-variation of ERLs and RTs, we introduce a new analysis procedure for estimating RT dependent ERLs (cf. Poli et al., 2010). For this purpose, vincentiles of single-trial EEG data were calculated. Basically, vincentiles are calculated by first sorting a data vector in increasing order and then separating it into the desired number of bins. Subsequently, the data are averaged within these bins. This is the standard procedure for response time data, which gives a relative precise description of the RT distribution (Ratcliff, 1979).

In the present study, RTs were collected from online event triggers in the EEG. Initially, RTs were sorted and the corresponding trial number was collected, also. Following this, the single-trial data of the ERL channels of interest (i.e., PO7/PO8) were sorted according to the RTs, using the collected trial numbers. Then, RTs and single-trial EEG data were binned (separately for each channel). In the present study, 10 bins were calculated. Finally, ERLs were computed for each bin (see Figure 3B) according to the procedure already described. This resulted in an averaged number of trials per bin of 39.26 (*SD* = 5.85) for the LUM condition, 33.60 (*SD* = 5.67) for the LOB condition and 41.29 (*SD* = 6.76) for the LOU condition.

In order to yield more reliable estimates of the RT bins, a time range of interest, i.e., where the ERL effects were most prominent, was selected for statistical quantification. More specifically, the time range from 160 to 510 ms was divided into 10 segments (35 ms each), from which the corresponding averages were computed (see Figure 6). For statistical quantification, all averaged segments in the derived time window of interest in the RT bins for each experimental condition (except the NoGo ORI condition), were tested against zero via non-parametric bootstrapping (Efron and Tibshirani, 1993) using 1000 bootstrap samples for each test. All derived *p*-values were adjusted by means of false discovery rate according to Benjamini and Yekutieli (2001). All statistics were calculated using GNU R (R Core Team, 2013).

## Results

### Behavioral data

**Figure 2:**
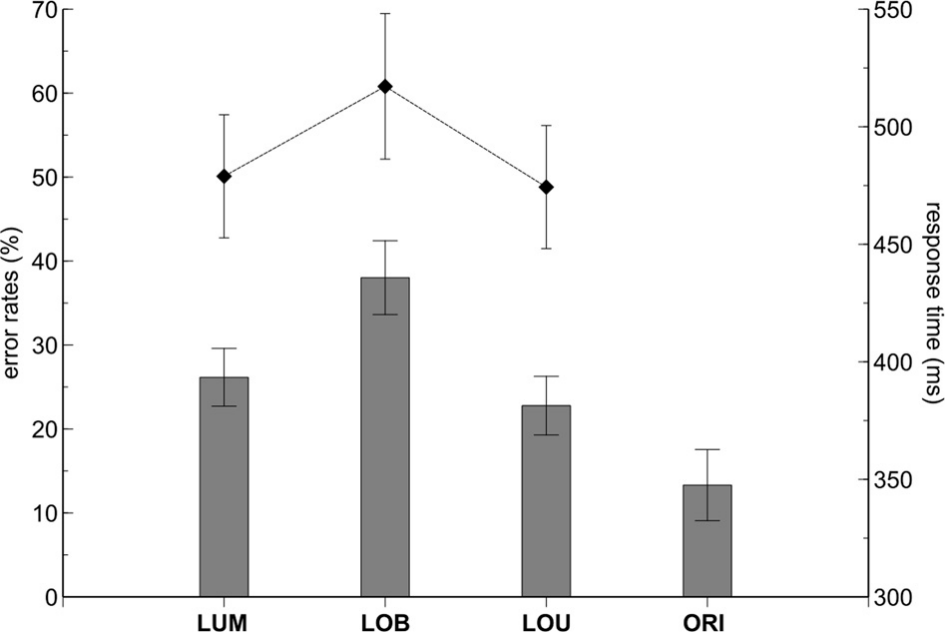
**Behavioral results**. The contralateral distractor condition (LOB) revealed higher error rates (bars) and response times (diamond shapes) compared to the remaining stimulus conditions. While both error rates and RTs did not differ between the LUM and LOU conditions, lower error rates were revealed in the No-Go ORI condition compared to all conditions containing a relevant luminance change (LUM, LOB, LOU). The error bars depict the standard error of the mean.

Error rates varied with change condition, *F*_(3,33)_ = 25.076, ε = 0.623, *p* < 0.001, η^2^_*p*_ = 0.695 (see Figure 2). Pairwise comparisons revealed higher error rates in the LOB condition compared to the LUM, *t*_(11)_ = 7.873, *p* < 0.001, *d* = 2.273, and LOU condition, *t*_(11)_ = 5.777, *p* < 0.001, *d* = 1.668. No statistically significant difference was observed between the LUM and LOU conditions, *t*_(11)_ = 1.627, *p* = 0.132, *d* = 0.47, while the ORI condition revealed the overall lowest error rates compared to the remaining change conditions (all *p*-values < 0.05). Error rates did not differ as a function of stimulus side, *F*_(1,11)_ = 0.954, *p* = 0.35, η^2^_*p*_ = 0.08, and also the change condition by side interaction was not statistically significant, *F*_(3,33)_ = 0.78, ε = 0.486, *p* = 0.517, η^2^_*p*_ = 0.066.

Comparable results were obtained on the basis of RTs. RTs varied with change condition, *F*_(2,22)_ = 44.178, ε = 0.682, *p* < 0.001, η^2^_*p*_ = 0.801, with highest values observed for the LOB condition (see Figure 1). Pairwise comparisons revealed higher RTs in the LOB condition compared to the LUM, *t*_(11)_ = 7.439, *p* < 0.001, *d* = 2.148, and LOU condition, *t*_(11)_ = 6.85, *p* < 0.001, *d* = 1.978. RTs in the LUM and LOU conditions did not differ significantly from each other, *t*_(11)_ = 1.477, *p* = 0.168, *d* = 0.427. Additionally, RTs tended to be faster for left luminance changes than for right luminance changes, *F*_(1,11)_ = 4.783, *p* = 0.051, η^2^_*p*_ = 0.303, while the change condition by side interaction was not significant, *F*_(2,22)_ = 0.466, ε = 0.867, *p* = 0.607, η^2^_*p*_ = 0.041.

### EEG data

#### Change-related positivity and N1pc

**Figure 3:**
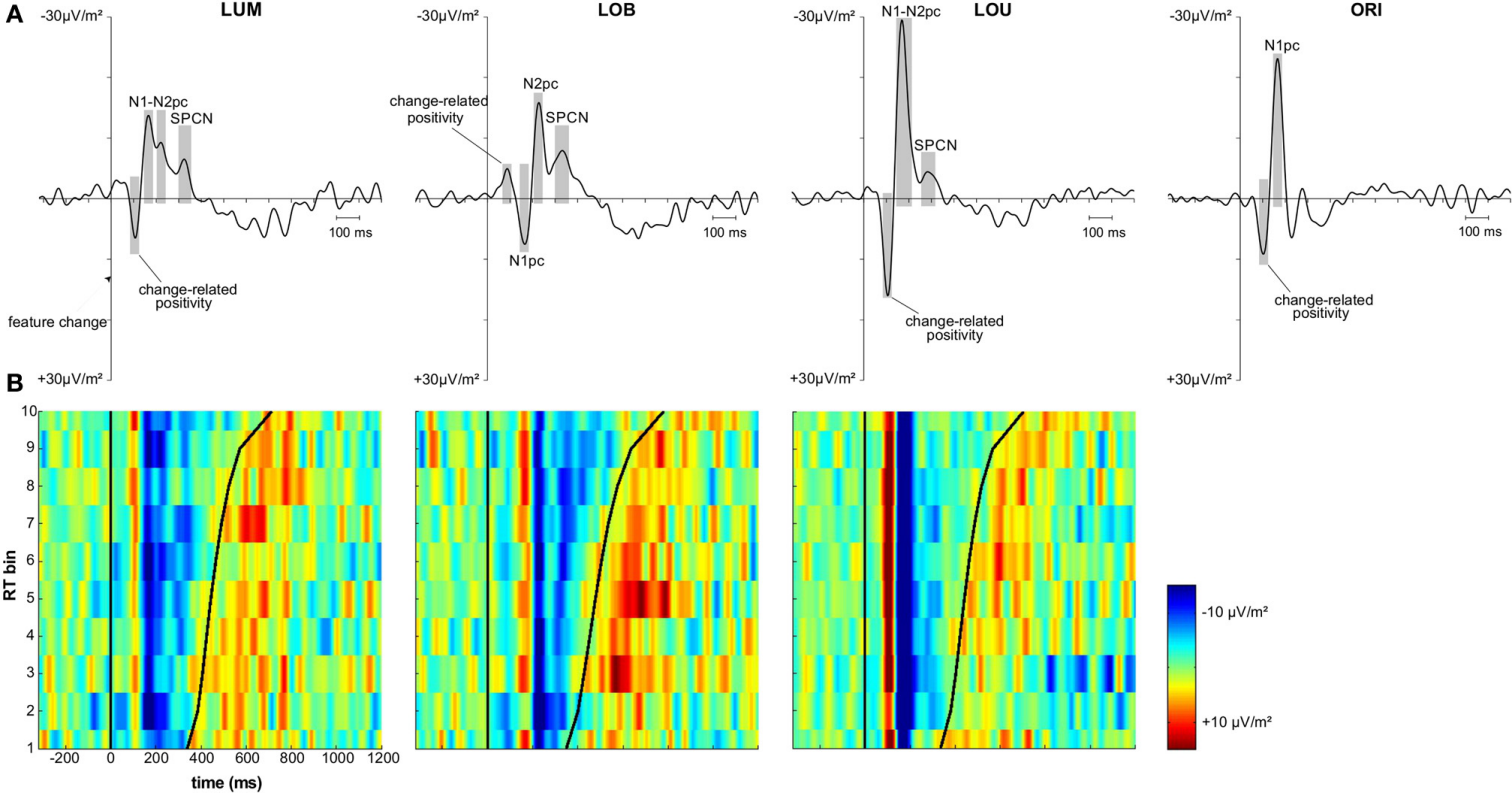
**Grand average and vincentized ERLs (PO7/PO8) for all change conditions. (A)** Depicts the grand average ERL data for the LUM, LOB, LOU, and ORI conditions. The components of interest (i.e., change-related positivity, N1pc, N2pc, and SPCN) were marked for each change condition. Both N2pc and SPCN were only analyzed in the target change conditions. **(B)** Depicts the vincentized ERLs for the LUM, LOB, and LOU conditions based on a color scale (negativity = blue, positivity = red) and the same time window also used for the grand average data. The bold black sinusoid line represents the mean response time (RT) across each bin. Change-related positivity, N1pc and N2pc were shown across all RT bins and revealed no consistent co-variation with RT based on both latency and amplitude of the components. However, the continuation of posterior contralateral negativity in the later time window (SPCN) was prolonged with increasing RTs in the LUM and LOB condition. The posterior asymmetry subsequent to response likely represented the lateralized sensory response to the manual key presses.

The first posterior deflection in the PO7/PO8 ERL locked to the change-relevant second stimulus display appeared in a time window from 70 to 120 ms subsequent to stimulus presentation (change-related positivity; see Figure 3A). This component revealed a positive deflection for the unilateral LUM condition, *t*_(11)_ = 5.235, *p* < 0.001, *d* = 1.511, LOU condition, *t*_(11)_ = 5.31, *p* < 0.001, *d* = 1.533, and ORI condition, *t*_(11)_ = 5.256, *p* < 0.001, *d* = 1.517. Additionally, mean amplitude varied with change condition, *F*_(2,22)_ = 14.751, ε = 0.753, *p* < 0.001, η^2^_*p*_ = 0.573. A larger positive deflection was observed for the LOU condition compared to the LUM, *t*_(11)_ = 4.344, *p* < 0.01, *d* = 1.254, and ORI conditions, *t*_(11)_ = 3.594, *p* < 0.01, *d* = 1.038, while the ORI condition in turn revealed a larger change-related positivity compared to the LUM condition, *t*_(11)_ = 2.493, *p* < 0.05, *d* = 0.72. Compared to these change conditions, the ERL deflection in this early time window was negative when luminance and orientation changes were presented contralateral to each other (LOB), *t*_(11)_ = −5.063, *p* < 0.001, *d* = 1.461. This further indicates that change-related positivity was less driven by the luminance change (i.e., the spatial reference) compared to the contralateral orientation change distractor.

Comparable results were observed for the subsequent N1pc component (150–190 ms after stimulus presentation; see Figure 3A). N1pc mean amplitude was negative for the unilateral LUM condition, *t*_(11)_ = −7.376, *p* < 0.001, *d* = 2.129, LOU condition, *t*_(11)_ = −9.536, *p* < 0.001, *d* = 2.753, and ORI condition, *t*_(11)_ = −9.98, *p* < 0.001, *d* = 2.881. Furthermore, N1pc mean amplitude varied with change condition, *F*_(2,22)_ = 26.23, ε = 0.895, *p* < 0.001, η^2^_*p*_ = 0.705. A larger negativity was observed for the LOU condition compared to the LUM, *t*_(11)_ = −7.154, *p* < 0.001, *d* = 2.065, and ORI conditions, *t*_(11)_ = −3.372, *p* < 0.01, *d* = 0.973, while the ORI condition in turn revealed a larger N1pc compared to the LUM condition, *t*_(11)_ = −3.844, *p* < 0.01, *d* = 1.1. The posterior ERL deflection in the N1 time window was inverted for the LOB condition. The resulting positivity differed significantly from zero, *t*_(11)_ = 3.669, *p* < 0.01, *d* = 1.059. Thus a higher N1 activation was shown contralateral to the irrelevant orientation change compared to the activation contralateral to the relevant luminance change (see Figure 3A).

#### N2pc and SPCN

In the current study, only the grand average ERL waveforms of the LUM and LOB conditions revealed both a negative peak in the N2 time window (200–250 ms subsequent to feature change presentation) and SPCN time window (at least 300 ms subsequent to feature change presentation). In the LOU condition, N2pc was merged to the strong N1pc complex and also SPCN appeared earlier compared to the LUM and LOB conditions (see Figure 3A). The ORI condition did not include a relevant stimulus and was therefore not included in the N2pc and SPCN analyses, because these components are associated with target selection and retention of relevant information in vSTM^2^.

**Figure 4:**
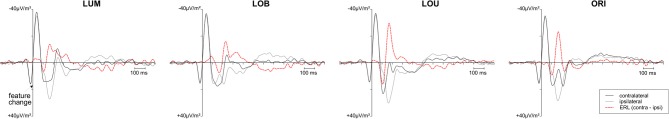
**Grand average posterior ERPs (PO7/PO8) contralateral and ipsilateral to the feature change**. In case of the luminance change conditions (LUM, LOB, LOU), the black curve represents the activation contralateral to the luminance change. For the ORI condition, the black curve represents the activation contralateral to the orientation change. The ERL (contralateral ipsilateral difference function) is plotted as a red dashed curve. Although an overlap of the P2 related to the first stimulus display and early sensory components related to the target display were revealed in the contralateral and ipsilateral ERPs, the ERL revealed a clear pattern of posterior asymmetries associated with lateralized target processing.

**Figure 5:**
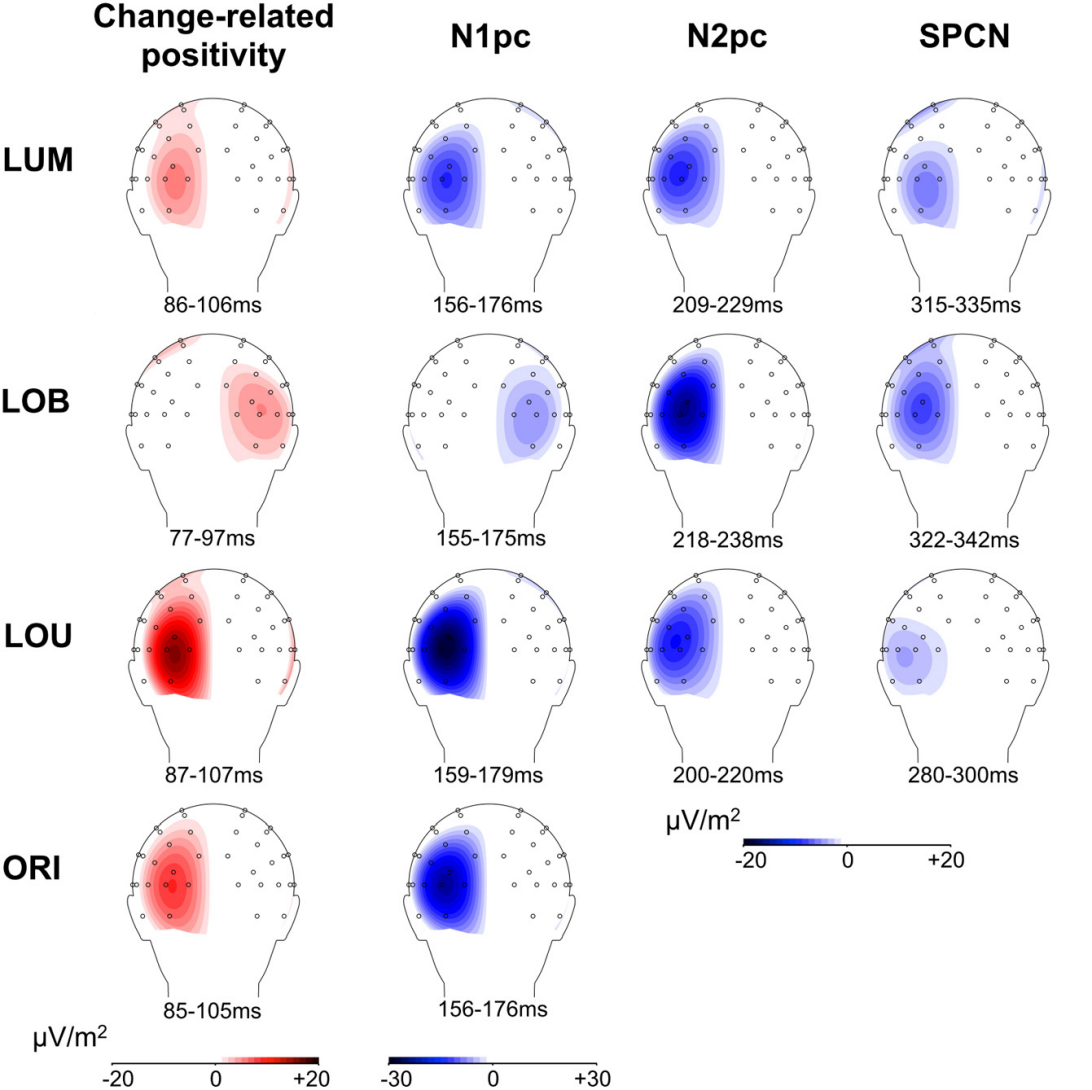
**Scalp topographies of posterior asymmetries for all change conditions**. For N1pc, N2pc, and SPCN, the higher negativity contralateral to the relevant luminance change compared to the negativity contralateral to the irrelevant stimulus is plotted on the left hemisphere, while an asymmetry toward the irrelevant stimulus is plotted on the right hemisphere (see LOB condition). The same logic applies to the change-related positivity. The depicted 20 ms time windows correspond to those used for statistical analyses and were oriented at the grand average ERL peaks. Because the LOU condition revealed no distinct ERL peak in the N2 range, a 200–220 ms time window was used to illustrate N2pc scalp topography.

Posterior ERLs revealed a negativity in the N2 time window (N2pc) for both the LUM, *t*_(11)_ = −3.428, *p* < 0.01, *d* = 0.99, and LOB condition, *t*_(11)_ = −6.017, *p* < 0.001, *d* = 1.737. This negativity was larger for the LOB compared to the LUM condition, *t*_(11)_ = −2.551, *p* < 0.05, *d* = 0.736. Furthermore, SPCN was reliably shown in the LUM condition, *t*_(11)_ = −2.331, *p* < 0.05, *d* = 0.673, LOU condition, *t*_(11)_ = −2.249, *p* < 0.05, *d* = 0.649, and LOB condition, *t*_(11)_ = −3.642, *p* < 0.01, *d* = 1.051. The mean amplitude oriented at the SPCN peak in the grand average did not differ as a function of change condition, *F*_(2,22)_ = 2.459, ε = 0.874, *p* = 0.117, η^2^_*p*_ = 0.183. Yet, while in the LOB condition SPCN was still reliably shown in the 350–400 ms interval, *t*_(11)_ = −2.904, *p* < 0.05, *d* = 0.838, a return to baseline was shown in the LOU, *t*_(11)_ = 0.205, *p* = 0.841, *d* = 0.059, and LUM condition, *t*_(11)_ = −0.521, *p* = 0.613, *d* = 0.15. For further information on the nature of the observed posterior ERLs, Figure 4 displays the posterior activation (PO7/PO8) contralateral and ipsilateral to the feature changes. Figure 5 shows the posterior scalp topographies of the described asymmetries (change-related positivity, N1pc, N2pc, SPCN) in time windows oriented at the peak of these components in the grand average.

#### Vincentized ERLs

Figure 3B shows the vincentized ERLs for the LUM, LOB, and LOU change conditions. The displayed time window was fitted to the grand averaged ERLs in Figure 3A (i.e., −320 to 1200 ms referred to the feature change onset). Positivity and negativity in the ERL were plotted in a color scale (negativity = blue, positivity = red). Obviously, the signal-to-noise ratio in the ERL of each RT was lower compared to the grand average ERL that was computed on basis of 10 times more trials. However, the components already described in the grand average ERL (see Figure 3A) were clearly reproduced in the vincentized data.

Both the LUM and LOU condition revealed an early positivity (change-related positivity) and subsequent negativity (N1pc) in the ERL with a constant latency across RT bins. While N2pc in the LOU condition was merged to N1pc for all RT bins, it was more distinctly represented in the LUM condition, but also showed no consistent variation between RT bins. The early deflection in the ERL of the LOB condition (change-related positivity) was not so consistently shown, possibly due to the generally lower amplitude compared to the remaining change conditions. Yet, both N1pc (positive deflection) and N2pc (negative deflection) were clearly represented in the vincentized ERLs of this change condition and also revealed a constant latency across RT bins.

**Figure 6:**
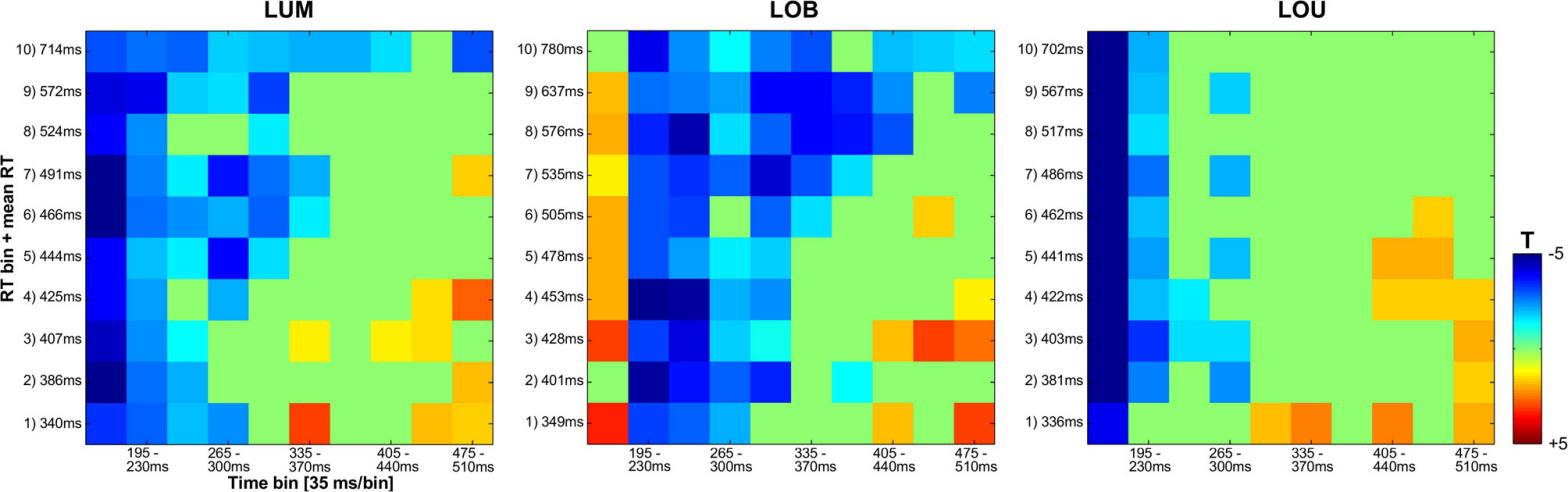
**Statistical analyses for the vincentized ERLs (PO7/PO8) based on bootstrap *t*-tests against zero (FDR corrected *p*-values)**. The reliable occurrence of posterior asymmetries was tested in 10 bins within an overall time window from 160 to 510 ms subsequent to the feature change(s). While a blue square represents negativity in the ERL that differed significantly from zero, a red square indicates a significant positivity in the posterior ERLs. Non-significant values are depicted in green. In the LUM and LOB conditions, a longer lasting negative difference from zero was shown for slower compared to faster responses. No comparable co-variation of RT and the continuation of posterior contralateral negativity was revealed for the LOU condition.

Regarding SPCN, both the vincentized ERL of the LOB condition and the LUM condition indicated a longer duration of posterior contralateral negativity with increasing RTs. This result pattern was further supported by the statistical analyses shown in Figure 6. When dividing the ERL into ten 35 ms time bins and testing these against zero for each RT bin, slow response RT bins exhibited a longer continuation of SPCN compared to faster RT bins. For example, the negative difference from baseline lasted until the fourth time bin (265–300 ms subsequent to change presentation) for the fastest 10% of responses in the LOB and LUM condition, but was prolonged until the tenth time bin (475–510 ms subsequent to change presentation) for the slowest 10% of responses. However, although SPCN was reliably shown in the grand average of the LOU condition, a comparable co-variation of SPCN continuation with RT was not supported by the vincentized ERLs of this change condition (see Figures 3B, 6).

Additionally, a positive ERL deflection subsequent to SPCN was shown for all change conditions containing a relevant luminance change (see Figure 3B). This component appeared subsequent to response and its topography revealed temporo-partietal maxima (parieto-occipital maxima were observed for SPCN; see Figure 5). This late asymmetry might reflect the lateralized sensory response to the button presses (referred to the tactile sense; cf. Wascher and Wauschkuhn, 1996).

## Discussion

The present study investigated the neural mechanisms that contribute to the detection of visual feature changes. Participants were instructed to locate a change of luminance in a fast succession of bilaterally presented bars that was randomly accompanied by an irrelevant change of orientation of the same or contralateral bar. Accuracy and RTs for change localization were impaired compared to the remaining luminance change conditions (LUM, LOU) when the task-irrelevant orientation change was presented contralateral to the target (LOB—contralateral distractor condition). Given the short presentation times and the short temporal offset of the two stimulus displays, the irrelevant change of orientation might have induced a lateral motion transient that distracted attention when relevant and irrelevant information were presented spatially separated and thus interfered with target processing (Beste et al., 2011; Schneider and Wascher, 2013). This was supported by prior studies revealing an increase of this distractor interference effect with increasing saliency of the orientation change or motion transient (Wascher and Beste, 2010; Schneider et al., 2012a,b).

We suggest that different processes are involved in the localization of relevant feature changes that can be studied by means of event-related lateralizations of the EEG (ERLs; i.e., contralateral—ipsilateral difference) referred to the bilateral stimuli. Furthermore, we propose a new vincentizing method based on these ERLs to more closely investigate the link between perceptual or attentional mechanisms and behavioral performance.

A first positive deflection in the ERL appeared with a latency of about 90 ms subsequent to the feature change(s) and was associated with the dishabituation of neurons encoding the feature change compared to those encoding the contralateral non-changing stimulus (Luck and Hillyard, 1994a; Verleger et al., 2012). The modulation of this initial response to feature changes between stimulus conditions supported this notion. When orientation and luminance changes appeared at the same positition (LOU), the saliency of change between the first and second stimulus display was larger compared to the single feature changes and thus also the dishabituation effect was stronger (see Figure 3A). We suggest that the observed early posterior contralateral positivity reflects a purely sensory response to feature changes of stimuli presented in fast succession and might be comparable to the change-related positivity observed in other studies (Kimura et al., 2005, 2006).

Also subsequent N1pc was associated with the sensory representation of the bilateral stimuli. However, prior studies revealed an attentional bias in favor of relevant signals already at this early stage of processing (see Schneider et al., 2012b). Comparable to the prior change-related positivity, N1pc was larger in amplitude for single orientation compared to single luminance changes and showed the highest posterior contralateral activation when both changes were presented at the same location (see Figure 3A). Furthermore, a positive ERL deflection in the N1 time window was shown in the LOB condition, indicating a stronger sensory representation of the irrelevant orientation change compared to the contralateral luminance change. On the other hand, N2pc is typically associated with the attentional selection of task-relevant visual information against distracting stimuli (Luck and Hillyard, 1994a,b; Eimer, 1996; Wascher and Wauschkuhn, 1996; Jolicoeur et al., 2008). The higher N2pc amplitude in the LOB condition compared to the remaining target conditions (LUM, LOU) indicates that an increased selective attentional bias in favor of the luminance change was required to compensate for the initially stronger representation of the irrelevant orientation change compared to the luminance change target (Wascher and Beste, 2010; Schneider et al., 2012a,b).

As laid down in the introduction, the later posterior contralateral negativity (SPCN) is associated with the maintenance of representations in vSTM subsequent to attentional selection until further cognitive operations with the fading neural representation of the stimulus material are completed (e.g., Prime et al., 2011; Mazza and Caramazza, 2012). Accordingly, a later SPCN return to baseline was observed in stimulus conditions that were associated with delayed responses to relevant signals compared to easier conditions (Robitaille and Jolicoeur, 2006). In a comparable way, a positive correlation was found between cue validity effects on SPCN onset latency and on RTs in a spatial cuing study (Brisson and Jolicoeur, 2008). Furthermore, Hickey et al. (2006) revealed a delayed posterior contralateral negativity in favor of a lateralized target when a contralateral distractor was presented compared to a no-distractor condition. This posterior asymmetry featured a latency comparable to SPCN in the current study^3^. However, to our best knowledge, the present study is the first to show that a co-variation of SPCN characteristics and RT is not only revealed between stimulus conditions, but also when visual stimulation remained constant (i.e., within conditions). As shown by the vincentized ERLs (see Figures 3B, 6), SPCN was prolonged with increasing time required to locate the relevant luminance change in the LOB and LUM conditions. This indicates a closer link between sustained vSTM representations and the speed of response selection and execution, because the modulation of SPCN continuation cannot be ascribed to varying complexities of the stimulus display (or exogenous factors).

Furthermore, Figure 5 indicates that the scalp distributions were similar for all described posterior asymmetries (change-related positivity, N1pc, N2pc, SPCN). Critically, despite the relatively late onset of SPCN at about 250–300 ms subsequent to change presentation, this component still revealed topographies with maxima over parieto-occipital cortex and was clearly distinguishable from more frontal asymmetries related to response preparation (see SPCN section in Figure 5). This suggests that the late posterior asymmetry was associated with a prolonged sensory representation of the relevant luminance change. The neural structures involved in early sensory processing of the stimulus material were also involved in later selective attentional mechanisms and got further re-activated to maintain a spatial stimulus representation in vSTM (Robitaille and Jolicoeur, 2006; Prime et al., 2011). Interestingly, errors in locating the relevant luminance change in the present paradigm were associated with a strong attentional distraction by the irrelevant orientation change and a lack of SPCN indicating a failure to encode the target in vSTM (Schneider et al., 2012a; Schneider and Wascher, 2013; see also: Prime et al., 2011). We therefore suggest that the re-activation of visual areas is required to form a stable percept of the presented relevant information that is actively maintained in form of spatial short-term memory representations (represented by SPCN) to evaluate and guide response selection and execution. This explains why SPCN was prolonged until participants were able to respond to the location of the relevant luminance change. Additionally, a sustained stable percept of relevant information might also play a role for aware perception when visual information is only briefly presented, because theories suggest that this state emerges when higher-level executive or mnemonic areas interact with lower-level representations to put the perceptual information in the context of current behavioral goals and needs (Duncan, 2001; Lamme, 2003).

However, no comparable co-variation of SPCN continuation and RT was shown in the LOU condition, although the occurrence of SPCN in the grand averaged data suggests that a lateralized representation of relevant information was at least frequently created in vSTM (see Figure 3). The relatively weak SPCN in the LOU condition might have led to a signal-to-noise ratio in the vincentized ERLs insufficient for depicting a reliable co-variation of SPCN continuation with RT. Additionally, the N1pc/N2pc complex in the LOU condition suggested an larger spatial representation of the relevant luminance change compared to the LUM and LOB conditions. This initially strong target representation in the visual system might have caused a more direct activation of the required response. Thus, the need for drawing on prolonged target representations in the visual system might have been reduced, leading to an independence of SPCN continuation and the speed of response-related mechanisms. In order to study this hypothesis, further research should investigate if comparable results can also be obtained in the LUM condition by scaling up the saliency of the relevant luminance change.

One additional point should be considered in further research. In the current study, only exogenous factors (the different stimulus conditions), but not the attentional setting of the observer was varied. Participants were instructed to focus on luminance changes during the whole experimental session. Concerning the generalizability of the current results, it should also be investigated if a comparable late posterior asymmetry co-variating with RTs can be observed when participants attend to orientation changes and have to ignore occasionally presented luminance changes.

In conclusion, the present study replicated that an irrelevant orientation change interfered with the processing of the luminance change target when it was presented at a contralateral position (see Wascher and Beste, 2010; Schneider et al., 2012b; Schneider and Wascher, 2013). While the early posterior contralateral positivity (i.e., change-related positivity) and negativity (i.e., N1pc) over visual areas were associated with the sensory response to stimulus change, the subsequent posterior negative ERL deflection in the N2 time window (i.e., N2pc) was related to the top-down allocation of attention to the relevant luminance change. Furthermore, we could show for the first time that the continuation of the later SPCN component associated with the maintenance of spatial target representation in vSTM varied with the time required for target localization within stimulus conditions. Also taking into account the similar scalp topographies for all analyzed posterior asymmetries, this suggests that information is re-processed within the visual system until a stable target percept is created in vSTM that can support response selection and the initiation of goal-directed behavior. Aware perception should critically depend on interactions between higher-level executive or mnemonic instances and such stable internal representations of visual information, especially when briefly presented relevant and irrelevant signals compete for neural representation within the processing system (see Desimone and Duncan, 1995).

### Conflict of interest statement

The authors declare that the research was conducted in the absence of any commercial or financial relationships that could be construed as a potential conflict of interest.

## Footnotes

^1^We referred to SPCN as a delayed N2pc in prior studies. However, the suggested underlying mechanisms were the same described in the current manuscript.

^2^The positive ERL deflections in the N2 and subsequent time windows of the No-Go ORI condition might in fact represent an active suppression of the orientation change distractor that is required for response inhibition.

^3^It can be argued whether the late N2pc in the study of Hickey et al. (2006) and SPCN in the current study indeed reflect the same mechanisms. In this context, the late posterior contralateral negativity might be associated with a re-focusing of attention on the target (Hickey et al., 2006). Comparable to the interpretation of SPCN effects in the current manuscript, this view also suggests an iterative processing of information in posterior visual areas.
